# Effectiveness of caffeine and blue-enriched light on cognitive performance and electroencephalography correlates of alertness in a spaceflight robotics simulation

**DOI:** 10.1038/s41526-023-00332-w

**Published:** 2023-12-19

**Authors:** Erin E. Flynn-Evans, Melanie Rueger, Andrew M. Liu, Raquel C. Galvan-Garza, Alan Natapoff, Charles M. Oman, Steven W. Lockley

**Affiliations:** 1 Division of Sleep and Circadian Disorders, Departments of Medicine and Neurology, Brigham and Women’s Hospital, 02115 Boston, MA USA; 2 Division of Sleep Medicine, Harvard Medical School, 02115 Boston, MA USA; 3 Fatigue Countermeasures Laboratory, Human Systems Integration Division, NASA Ames Research Center, Moffett Field, CA 94035 USA; 4 Human Systems Laboratory, Massachusetts Institute of Technology, Cambridge, MA 02139 USA

**Keywords:** Translational research, Outcomes research

## Abstract

Human cognitive impairment associated with sleep loss, circadian misalignment and work overload is a major concern in any high stress occupation but has potentially catastrophic consequences during spaceflight human robotic interactions. Two safe, wake-promoting countermeasures, caffeine and blue-enriched white light have been studied on Earth and are available on the International Space Station. We therefore conducted a randomized, placebo-controlled, cross-over trial examining the impact of regularly timed low-dose caffeine (0.3 mg per kg per h) and moderate illuminance blue-enriched white light (~90 lux, ~88 melEDI lux, 6300 K) as countermeasures, separately and combined, in a multi-night simulation of sleep-wake shifts experienced during spaceflight among 16 participants (7 F, ages 26–55). We find that chronic administration of low-dose caffeine improves subjective and objective correlates of alertness and performance during an overnight work schedule involving chronic sleep loss and circadian misalignment, although we also find that caffeine disrupts subsequent sleep. We further find that 90 lux of blue-enriched light moderately reduces electroencephalogram (EEG) power in the theta and delta regions, which are associated with sleepiness. These findings support the use of low-dose caffeine and potentially blue-enriched white light to enhance alertness and performance among astronauts and shiftworking populations.

## Introduction

The success and effectiveness of spaceflight operations depends on the ability of crewmembers to maintain a high level of cognitive performance and vigilance over long durations. Mission schedules often require astronauts to shift their sleep-wake timing from a standard ‘day’ including occasional ‘slam-shifts’, whereby the sleep-wake and work cycles are inverted, inducing circadian misalignment and acute sleep deprivation^1,2^. These episodes of acute sleep loss occur on a background of prolonged chronic sleep deficiency; several studies have demonstrated that habitual sleep duration is shorter during spaceflight relative to pre- and post-flight, with astronauts sleeping only six hours a night in space, on average^1,3,4^. These sleep patterns are of particular concern given that such sleep deficiency has been associated with performance impairment including reduced reaction time^5^ and a few catastrophic outcomes during spaceflight operations, including a collision between the Progress re-supply ship and the Mir Space Station in 1997^6^ and impaired decision-making by support personnel in advance of the Shuttle Challenger accident^7^. On Earth, short sleep duration, circadian misalignment and long work hours have repeatedly been linked to cognitive impairment in other professions with highly trained and highly motivated personnel^8^, including an increase in attentional failures^9^, actual and near-miss medical errors^10^ and fatigue-related motor vehicle crashes^11^ among medical residents, and poor health outcomes in shiftworkers^12–15^.

While measures should be taken to provide crewmembers with stable sleep schedules in an optimized environment, direct, safe, wake-promoting countermeasures are often needed to minimize the safety risk due to sleepiness when sleep and work patterns are not optimal. Two safe, wake-promoting countermeasures are feasible for use on future space vehicles and are currently available on the International Space Station (ISS). Caffeine, the most widely used performance-enhancing drug, is provided during spaceflight in the form of pills and liquid coffee although its use is currently unregulated. Numerous studies have confirmed the utility of caffeine as a countermeasure against the effects of sleepiness on certain types of neurobehavioral performance^16–20^ including for up to 28 hours awake when administered chronically in low doses (0.3 mg per kg per h)^21^.

Another stimulant that is available to the ISS crew is light. Light is both an acute stimulant^22,23^ and the most powerful time cue for resetting the circadian pacemaker and the rhythms it controls, including the sleep/wake cycle^24^. These ‘non-visual’ effects of light are primarily mediated by a novel non-rod, non-cone photoreceptor in the eye that is most sensitive to short-wavelength light (*λ*_max_ ~ 480 nm)^25–28^. Blue and blue-enriched light have been used as countermeasures to enhance alertness and performance in laboratory^25,26,29,30^ and field^31–33^ studies. Beginning in 2016, the fluorescent lighting aboard ISS was gradually replaced with a programmable multi-LED lighting system that can provide a light program with different spectra and intensities at different times of day in order to maximize alertness and performance (for example, when working overnight) or to minimize alertness (for example, in the hours before scheduled sleep) as required^34^.

We therefore hypothesized that caffeine and/or blue-enriched white light would be effective countermeasures for improvement of subjective and objective correlates of alertness and performance during episodes of circadian misalignment and chronic sleep restriction. To test this hypothesis, we conducted a within-subject study with three randomized countermeasures, consisting of caffeine and blue-enriched light alone, and in combination, to assess alertness and performance in a 13-day ISS analogue simulation of a ‘slam shift’ on a background of chronic sleep restriction.

## Results

### Participants and protocol

**Fig. 1:**
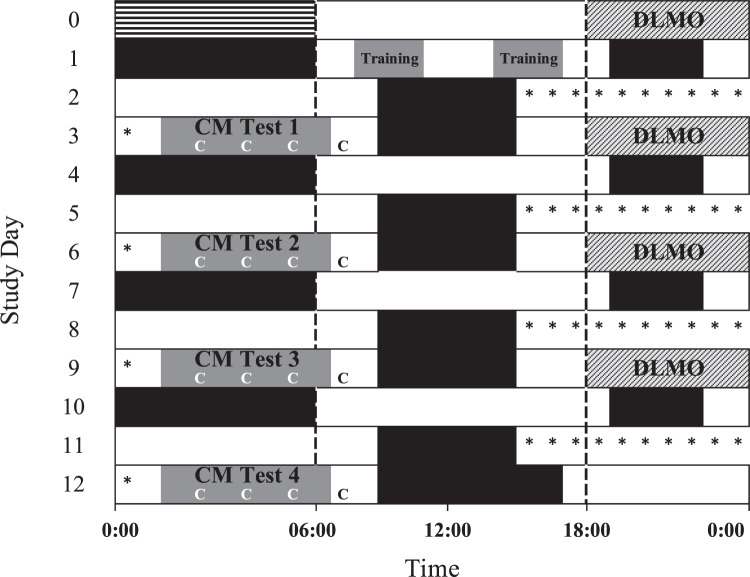
Inpatient study protocol. Participants were admitted to the laboratory in the morning of study day 0. The striped bar reflects habitual sleep time at home, black bars indicate sleep in the laboratory. Dark gray bars indicate the timing of the robotics training and testing sessions. Hatched bars indicate the timing of dim light melatonin onset (DLMO) sampling. The timing of the robotics simulation occurred during each of the gray sessions labeled “CM Test.” The timing of the cognitive test batteries are indicated with a “C.” Asterisks indicate the timing of placebo or caffeine pill administration, which continued through the robotics sessions until 2 hours before bedtime. The two gray bars labeled “training” reflect the in-laboratory robotics training simulations on Day 1.

We measured cognitive performance, subjective sleepiness ratings and objective EEG correlates of alertness hourly in healthy astronaut-aged participants (*n* = 16, 7 F, 26–55 years) during four separate 6-hour overnight robotics and cognitive performance task batteries including the Visual Analogue Scale (VAS), the Karolinska Sleepiness Scale (KSS), the Digit Symbol Substitution Task (DSST), the 10-minute visual version of the Psychomotor Vigilance Test (PVT), and the Karolinska Drowsiness Test (KDT), under four conditions; standard white light (94 lux, 71 melEDI lux, 4700 K) plus placebo (baseline); A) 4700 K light plus chronic low-dose caffeine; B) moderate intensity blue-enriched (94 lux, 88 melEDI lux, 6300 K) white light plus placebo; and C) blue-enriched light combined with caffeine, in a within-subject design (Fig. 1; Supplemental Fig. 1). Participants were randomized into one of three countermeasure orders; BCA (*n* = 6), ACB (*n* = 6), CBA (*n* = 4). The light source for all experimental exposures was a multi-LED Solid State Lighting Module (SSLM), developed as a prototype for the LEDs that replaced the fluorescent lighting aboard the International Space Station^33^. The moderate intensity blue-enriched 6300 K white light was selected based on the enhanced melatonin suppression, circadian phase shifting and alerting response to blue light^25,26,35–37^. The chronic, low-dose caffeine regime was chosen (0.3 mg per kg per h administered from waketime until 2 hours prior to bedtime) based on its demonstrated alerting effects over 28 hours of continued wakefulness^21^. Analysis of cognitive performance was restricted to performance tests conducted during the biological night, defined as those occurring after plasma dim light melatonin onset (DLMO) time, measured in each individual prior to each condition (Fig. 1), given the potentially confounding effects of the spontaneous improvement in performance due to the ‘wake maintenance zone’ (WMZ) immediately prior to DLMO^38^.

### Cognitive performance

**Fig. 2:**
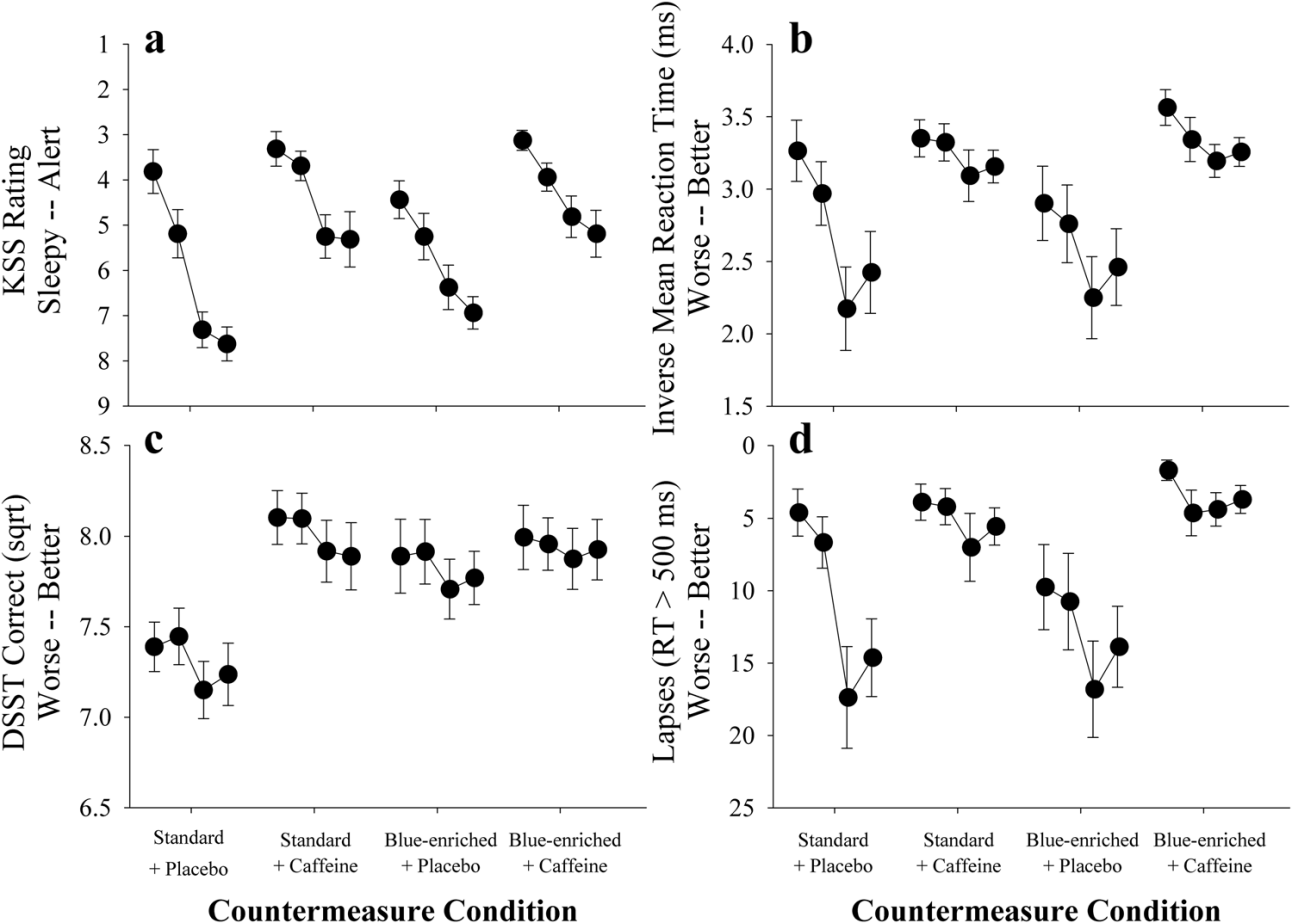
Response to countermeasures. Each panel shows the results of the four cognitive test sessions that occurred during the countermeasure evaluation block. **a** Karolinska Sleepiness Scale (KSS); **b** Mean inverse reaction time on the Psychomotor Vigilance Task (PVT); **c** Square root of the mean number of correct responses on the digit symbol substitution task (DSST); **d** Mean number of lapses on the PVT. All values shown +/− standard error.

The data from the baseline condition (4700 K plus placebo) showed that, as intended, participants were impaired following a week of maintaining a 6-hour sleep schedule (Fig. 2). The average PVT reaction time for the baseline data collection block was 567 ms (±476), speed (inverse mean) of 2.7 (±0.9), with an average of 12 (±9) lapses >500 ms, which is considerably above the mean range of 200–270 ms, with <10 lapses per test, observed among rested individuals^39–42^.

PVT reaction time (inverse mean; response speed) was significantly faster during the two caffeine conditions (4700 K + caffeine, mean speed = 3.2 ± 0.5 SD, *β* = 0.59 *p* < 0.01; 6300 K + caffeine, mean speed = 3.3 ± 0.4, SD, *β* = 0.67 *p* < 0.01), but not in the 6300 K + placebo condition, mean speed = 2.6 ± 1.0 SD (*β* = 0.09, *p* = 0.42), compared to the baseline condition (Fig. 2). Lapses (mean reaction times > 500 ms) showed a similar pattern (4700 K + caffeine, lapses = 5.1 ± 5.1 SD, *β* = −6.6, *p* < 0.01; 6300 K + caffeine, lapses = 3.6 ± 3.8 SD, *β* = −8.1, *p* < 0.01), but not in the 6300 K + placebo condition, lapses = 11.7 ± 9.5 SD (*β* = 1.1, *p* = 0.17). Subjective visual analogue scale (VAS, arcsine transformed) ratings of alertness showed the same pattern, with participants rating sleepiness as significantly better during the 4700 K + caffeine (mean 64.0 ± 15.3 SD, *β* = 0.19, *p* < 0.01) and 6300 K + caffeine (mean 65.8 ± 10.4 SD, *β* = 0.2, *p* < 0.01) conditions and no difference during 6300 K + placebo (mean 51.0 ± 15.5, *β* = 0.05, *p* = 0.27) compared to the 4700 K + placebo condition (mean 46.0 ± 15.7 SD). The KSS showed the same pattern (4700 K + placebo, 6.0 ± 1.2 SD; 4700 K + caffeine, 4.5 ± 1.5 SD, *β* = −1.5, *p* < 0.01; 6300 K + caffeine, 4.3 ± 1.2 SD, *β* = -1.7, *p* < 0.01), with no differences in the 6300 K + placebo condition (5.8 ± 1.4 SD, *β* = −0.1, *p* = 0.69; Fig. 2). The square root of the number of correct responses on the DSST was significantly faster during all three experimental conditions compared to the baseline condition (4700 K + placebo, √*correct* = 7.3 ± 0.6, 4700 K + caffeine, √*correct* = 8.0 ± 0.6, *β* = 0.7, *p* < 0.01; 6300 K + caffeine, √*correct* = 8.0 ± 0.6, *β* = 0.6, *p* < 0.01; 6300 K + placebo, √*correct* = 7.8 ± 0.6, *β* = 0.5, *p* < 0.01; Fig. 2).

### Electroencephalogram power

**Fig. 3:**
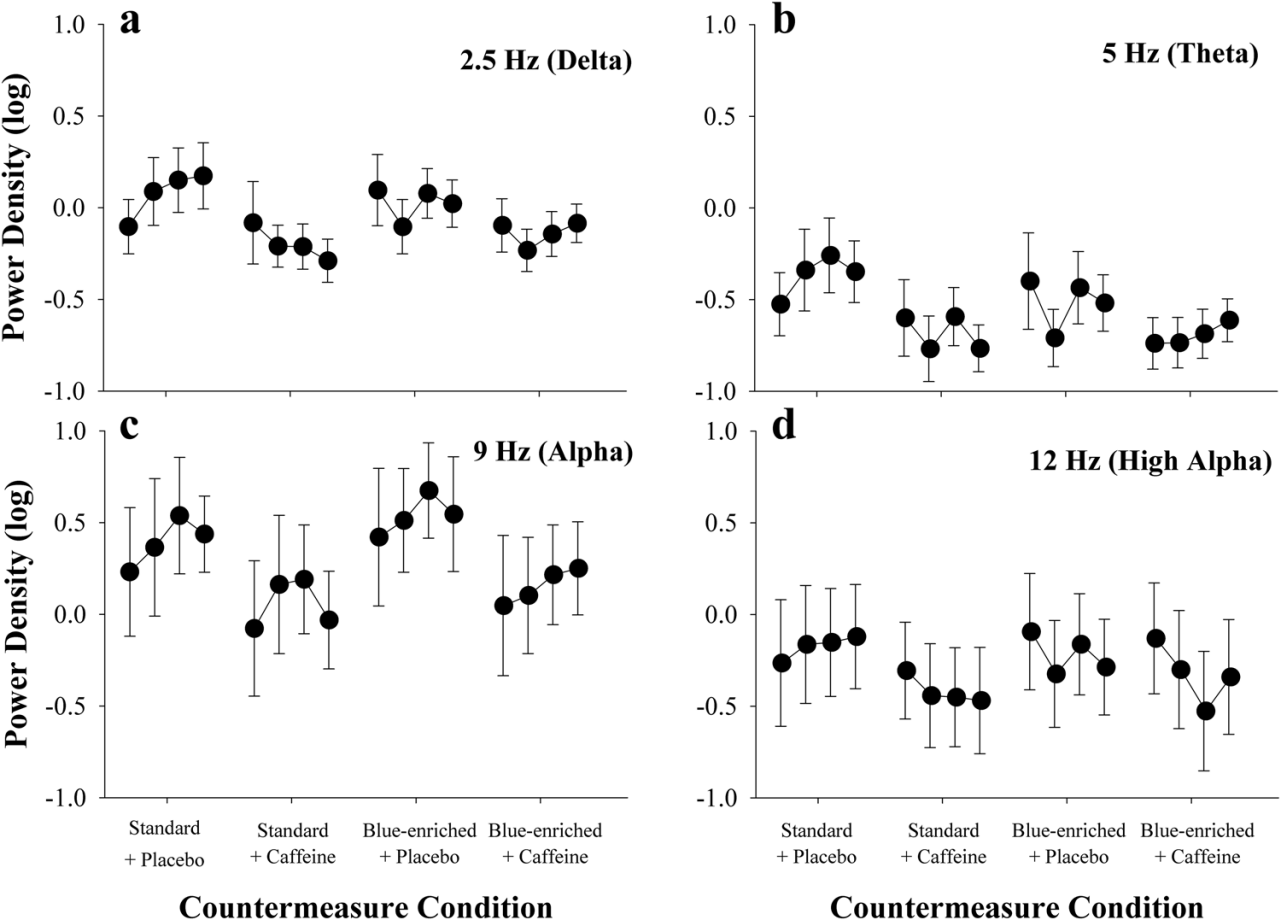
Power density of four selected EEG frequencies during the Karolinska Drowsiness Test (KDT). Each panel shows the results of the four KDT sessions that occurred during the countermeasure evaluation block.

Waking EEG recordings during 3-minute KDTs performed between the robotics task were used to derive objective correlates of alertness based on pre-determined frequency bands. Delta power (evaluated at 2.5 Hz), considered a marker of sleepiness, was significantly reduced in the two caffeine conditions (4700 K + caffeine, *β* = −0.3, *p* < 0.01; 6300 K + caffeine, *β* = −0.2, *p* < 0.01; Fig. 3) and was moderately but non-significantly reduced in the 6300 K + placebo condition (*β* = −0.1, *p* = 0.23) relative to baseline. Theta power (5.0 Hz) was significantly reduced in all three countermeasure conditions as compared to baseline, indicating greater alertness (4700 K + caffeine, *β* = −0.3, *p* < 0.01; 6300 K + caffeine, *β* = −0.3, *p* < 0.01; 6500 K + placebo, *β* = −0.2, *p* = 0.01). Alpha power (9 Hz) was significantly reduced in the 4700 K + caffeine and 6300 K + caffeine conditions (both *p* < 0.01, *β* = −0.3 and −0.2, respectively) and did not differ in the 6300 K + placebo condition (*β* = 0.2, *p* = 0.05). High alpha power (12 Hz) was significantly reduced during the caffeine treatments relative to baseline (4700 K + caffeine, *β* = −0.2, *p* < 0.01; 6300 K + caffeine, *β* = −0.2 *p* < 0.01), but was not significantly reduced in the 6300 K + placebo condition (*β* = −0.1, *p* = 0.11). Effect sizes by outcome for each condition relative to placebo are shown in Table 1.

**Table 1:** Effect sizes between the robotics, cognitive and electroencephalography (EEG) outcomes measures by study condition relative to placebo.

	6300 K + Caffeine	4700 K + Caffeine	6300 K + Placebo
***Robotics***			
Capture Task (mean 1/RT)	0.61	0.67	0.18
Position Task (mean 1/RT)	0.62	0.69	0.11
Autosequence Task (mean 1/RT)	0.59	0.70	0.12
Side task (Missed detections)	−0.60	−0.60	−0.23
***Cognition***			
PVT (mean 1/RT)	0.76	0.61	−0.09
PVT (lapses > 500 ms)	−1.08	−0.83	−0.03
KSS	−1.25	−0.98	−0.14
Digit Symbol Substitution Test (√*correct*)	1.03	1.03	0.74
VAS (Alertness)	1.26	1.03	0.28
***Electroencephalography***			
Delta power (log [2.5 Hz])	−0.37	−0.53	−0.09
Theta power (log [5 Hz])	−0.51	−0.49	−0.20
Alpha power (log [9 Hz])	−0.18	−0.34	−0.50
High alpha power (log [12 Hz])	−0.18	−0.22	−0.07

Better performance in the countermeasure conditions relative to placebo are indicated by positive values for all robotics and cognition outcomes, except for the side task, where negative effect sizes indicate fewer missed detections, Psychomotor Vigilance Task (PVT) lapses, where lower scores indicate fewer lapses, and the Karolinska Sleepiness Scale (KSS), where lower scores indicate lower sleepiness ratings. All EEG outcomes indicate lower power in the countermeasure conditions relative to placebo.

*RT* reaction time, *ms* milliseconds, *Hz* hertz, *VAS* visual analogue scale ranging from “sleepy” to “alert” on a 100 mm scale.

### Sleep architecture

Sleep architecture following the intervention days varied by countermeasure condition. Percentage of wake during the sleep episode was not different from baseline (mean 8.6% ± 5.8% SD) in the 6300 K + placebo condition (mean 8.9% ± 7.8% SD) but increased in each of the caffeine conditions (4700 K + caffeine, mean 22.1% ± 17.7% SD, *p* < 0.01 and 6300 K + caffeine, mean 26.4% ± 20.5% SD, *p* < 0.01). Percentage of stage 1 sleep did not differ in any of the countermeasure conditions relative to baseline (4700 K + placebo, 6.2% ± 4.2% SD; 4700 K + caffeine, 6.4% ± 2.8% SD, *p* = 0.78; 6300 K + caffeine, 7.2% ± 2.4% SD *p* = 0.17; 6300 K + placebo condition, 5.0% ± 2.8% SD *p* = 0.08). Percentage of stage 2 sleep did not differ from baseline (mean 39.7% ± 7.4% SD) in the 4700 K + caffeine (36.9% ± 10.0% SD, *p* = 0.32) or 6300 K + placebo condition (39.5% ± 6.6% SD, *p* = 0.95) but was significantly reduced in the 6300 K + caffeine condition (33.5% ± 11.8% SD, *p* = 0.04). Percentage of slow wave sleep was significantly reduced in all three conditions relative to baseline (4700 K + placebo, 24.9% ± 4.4% SD; 4700 K + caffeine, 14.5% ± 5.8% SD, *p* < 0.01; 6300 K + caffeine, 15.5% ± 6.0% SD *p* < 0.01; 6300 K + placebo condition, 21.4% ± 7.0% SD *p* = 0.03). Finally, the percentage of REM in the sleep episode was significantly increased in the 6300 K + placebo condition (24.9% ± 6.3% SD *p* = 0.03), but the caffeine conditions were not significantly different from baseline (4700 K + placebo, 20.4% ± 6.3% SD; 4700 K + caffeine, 20.0% ± 8.2% SD, *p* = 0.79; 6300 K + caffeine, 17.3% ± 8.2% SD *p* = 0.06).

## Discussion

In a simulated ISS mission schedule, administration of low-dose, regularly timed caffeine was able to counteract the alertness and performance decrements induced by chronic sleep loss and circadian misalignment. Caffeine improved alertness and performance in all measures, including standard cognitive tests and secondary robotics tasks. Exposure to moderate intensity blue-enriched white light showed similar but smaller improvements in some measures including improved performance on the DSST, and reductions in EEG theta power, high levels of which are associated with sleepiness. The combination of caffeine and blue-enriched light exhibited the largest effect sizes for some outcomes, suggesting a potential positive effect of using both stimulants together.

Caffeine is the most widely used wake-promoting drug worldwide. Regularly timed administration of low-dose caffeine (equivalent to only 21 mg per h in a 70 kg person, or less than half a standard soda or tea) elicited sustained high performance on the PVT during a repeated ‘slam shift’ involving a sudden transition to night work following a week of chronic sleep restriction of six hours. It also improved performance on the DSST and on subjective ratings of sleepiness. These findings confirm and extend an earlier laboratory trial that demonstrated that caffeine is an effective countermeasure when used strategically in this way^21^ and suggests that astronauts and individuals required to work during the biological night, under chronic sleep restriction, would benefit from a strategic, low-dose caffeine administration protocol, notwithstanding inter-individual differences in caffeine sensitivity^43^.

While caffeine is clearly a potent countermeasure to improve alertness and performance on a variety of tasks, we confirmed the findings of others^44^, demonstrating that caffeine interferes with sleep. Despite chronic sleep restriction, participants in our study experienced significantly more awake time and significantly less slow wave sleep following caffeine administration compared to baseline. In practice, in order to minimize the negative impact of caffeine on sleep, low-dose caffeine could be used until half-way through an overnight shift, with the remaining circulating caffeine helping to maintain alertness for the next several hours. Limiting use to work days may also prevent tolerance and reduced effectiveness with chronic use.

There are few data available on the use of caffeine by astronauts during spaceflight. In post-flight interviews, 75% of astronauts reported use of alertness medications during their mission, including caffeine pills or modafanil^45^ and ISS crewmembers reported using caffeine on more than 90% of days before and during long duration missions, regardless of whether or not they were circadian misaligned^2^. Habitual, rather than strategic, caffeine use and the presumably near-constant presence of such a powerful performance enhancing drug likely masks the true extent of sleepiness due to chronic sleep loss and circadian misalignment during space flight and analogue missions. We therefore have little information on what would happen to sleepiness and performance in operational settings if caffeine became unavailable during a long-duration mission, and plan for that contingency. Our current study suggests that the impact would be quite profound—performance was worst under the placebo condition with standard lighting—and likely of concern for the performance and safety of the crew. We encourage NASA to conduct analogue studies without access to caffeine to measure intrinsic performance levels under those conditions.

Light is both an acute stimulant and the most powerful time cue for resetting the circadian clock. The wavelength, intensity, timing, pattern and duration of the light stimulus determine the magnitude of the alerting and phase shifting effects. The light source that we used was broad-spectrum white light, to permit good vision^33^, but was tuned to enhance energy in the short-wavelength blue range in order to preferentially stimulate melanopsin-containing retinal ganglion cells (Supplemental Fig. 1). The human circadian pacemaker is most sensitive to short-wavelength light in the ~480 nm range^25,46^ due to stimulation of the melanopsin-containing retinal ganglion cells that project to the suprachiasmatic nucleus in the hypothalamus^47^ although the photopic visual system (λ_max_ 555 nm) has also been shown to contribute to light-induced alerting and phase shifting albeit sub-maximally^35^. Short-wavelength sensitivity has been demonstrated for the acute effects of light on subjective alertness ratings, auditory performance, heart rate, and thermoregulation^28,29,48,49^ and for the change in power of EEG frequencies that indicate a more alert state^25–27^.

We have previously demonstrated that exposure to monochromatic blue light (460 nm) preferentially enhanced EEG power in the high-alpha frequency range (10.5–11.5 Hz) and suppression of delta/theta power (0.5–5.5 Hz)^25,26^ and others have shown similar responses during exposure to blue-enriched polychromatic light^50,51^. Although we found that the blue-enriched light condition was able to reduce theta power, we did not observe an enhancement in the high alpha region of the EEG. Cognitive responses were also mixed; blue-enriched white light improved information processing performance on the DSST but did not improve PVT performance. These differences are likely due to the fact that our light source was at a lower illuminance than in prior studies. In addition, our study differs from prior studies in that we sleep restricted our participants to six hours of sleep per night for the week prior to the baseline condition, followed by repeated slam shifts in order to evaluate an operationally relevant schedule. As the baseline condition was always first, participants would have accumulated a substantially greater chronic and acute sleep debt by the time of the first countermeasure exposure relative to baseline. It is likely that a stronger light stimulus was required to improve alertness during subsequent slam shifts^52^.

Unlike caffeine, the blue-enriched white light alone had little impact on sleep architecture. The amount of wake in the sleep episode following light exposure was not different from the baseline condition and the percentage of REM sleep was increased. Slow wave sleep was significantly, but only modestly reduced following exposure to blue-enriched light. These findings provide important considerations for identifying appropriate countermeasures for different operational scenarios.

This study was designed to evaluate a countermeasure regime under conditions mimicking those experienced by astronauts on the ISS^1^. We studied participants from the astronaut corps age range, with bachelor-level education and good physical health, simulated prior sleep-wake history^1^ and scheduled sleep-wake and work shift changes similar to those used in space^2^, employed LED lighting prototypes and geometry similar to those currently installed aboard the ISS^34^, tested performance using a high fidelity robotics simulator used in initial astronaut training (Liu et al.,^53^ accompanying manuscript), and assessed cognitive function using a subset of the measures currently used by NASA^54^. Notwithstanding these efforts, we did not study active astronaut crews, and it has been argued that populations who choose to engage in shiftwork, particularly in specialist, high-stress occupations, are self-selected based on their potential resilience to sleep- and circadian-related cognitive impairment. While studies have demonstrated inter-individual differences such that some individuals are indeed able to sustain performance over the course of extended wakefulness^42,55^, and during complex robotics operations^56^, such resilience appears to be limited to specific aspects of cognitive function, meaning that exceptional individual performance on a test of one facet of cognitive function does not assure such performance will be achieved in other cognitive domains^57^. Similarly, not all outcomes were impacted to the same degree by each countermeasure which may have been due to inter-individual differences in performance or differential responses of the task to the countermeasures, or both. It is also possible that not all participants achieved the same quality of sleep throughout the protocol. Notably, performance was still poorer than that typical of rested individuals (Fig. 2^39–42^; in all countermeasures. We attempted to reduce the variability in response to these countermeasures by placing the baseline condition first and studying a three, rather than six, randomization orders. It is possible that these methodological choices may have influenced our results. Overall, our findings suggest that these countermeasures can restore some, but not all of the performance deficit that arises from chronic sleep loss and circadian misalignment and may affect individual crewmembers differently. It is therefore imperative that schedules are designed using sleep and circadian principles to facilitate alertness as well as allow for adequate recovery sleep between episodes of extended duration work, as sleepiness countermeasures alone will likely not alleviate all sleepiness risk.

We designed our blue-enriched white light treatment countermeasure to meet several requirements necessary for deployment in spaceflight. Although we selected lighting that would appear visibly white to the participants, two of the experimenters (E.E.F. and M.R.) were aware of the lighting condition during administration. Our statistician (A.N.) had no knowledge of the conditions until after the analysis was complete, however. Our goal was to select a passive light-exposure regime that would stimulate the maximum biological effect with minimal energy expenditure, while also providing suitable task lighting for completion of a simulated spaceflight task, which therefore required white light^34^ rather that narrow bandwidth blue-light as a passive countermeasure^58^. In hindsight, although we set the light level based on 50% of the maximal alerting response to 4100 K white light overnight^27^, the illuminance level chosen was likely too low. A passive, blue-enriched light exposure (8000 K, ~180 lux, 170 melEDI lux), combined with exercise, during breaks was able to significantly improve alertness and performance during night shifts at NASA Mission Control^31^. Similarly, blue-enriched lighting exposure (~5000 K, up to ~380 lux and 380 melanopic EDI lux) installed in the breakroom and nursing stations of an ICU for use overnight significantly reduced the rate of serious medical errors^59^. Furthermore, when combined with the light emitted from the robotics simulator screens, the different in melanopic EDI lux between the conditions was modest (Supplemental Fig. 1). It is therefore likely that it would be possible to improve the efficacy of the blue-light countermeasure treatment with a higher melanopic illuminance, which can be achieved by the Solid-State Lighting Assemblies (SSLA) now installed on ISS^60^. A recent expert consensus statement recommended 250 melanopic EDI lux as the minimum level to enhance alertness and circadian entrainment^61^.

Our findings have important implications for countermeasure treatment regimes for astronauts and shiftworkers alike. Astronauts have been shown to have reduced vigilant attention on a reaction time test (reaction self-test) following nights with less than five hours of sleep^5^. Our findings suggest that caffeine or the combination of caffeine and light are tools that can help restore some of the deficits arising from such chronic sleep loss. Although both caffeine^21^ and blue-enriched light^62^ have independently been shown to be effective countermeasures to improve alertness and performance during night work and circadian misalignment, caffeine and blue-enriched light together may be more effective than either countermeasure alone. When delivered together, these countermeasures have been shown to improve performance together across a range of tasks in other studies using higher^63^ and similarly low doses of caffeine and blue-enriched light^64,65^. Further refinement of dose and timing is required to optimize these benefits for operational use.

## Methods

### Participants

Healthy research volunteers (*n* = 17, 7 F, mean age ± SD: 37.1 ± 8.1 years), aged 26-55 years were enrolled in a 13-day inpatient study at the Intensive Physiologic Monitoring Unit (IPM), Brigham and Women’s Hospital (BWH; Boston, MA) between November 2010 and June 2012. Informed consent was obtained from all participants, and research procedures were approved by the Institutional Review Board at BWH and were in compliance with HIPAA regulations and the Declaration of Helsinki. Participants were required to hold at least a bachelor’s degree and be within the astronaut corps training age range. One participant (3163 V) had to leave the study early due to a family emergency, resulting in a total of sixteen subjects with complete data sets (7 F, mean age ± SD: 37.31 ± 7.74 years).

Physical health was assessed by medical history, physical examination, blood biochemistry and hematology, and electrocardiogram. Mental health was evaluated by interview with a staff psychologist/psychiatrist. Normal sight, including the absence of color-blindness, was confirmed by an ophthalmologic examination before and after the study. Sleep and circadian-rhythm disorders were exclusionary. Participants were instructed to maintain a self-selected, regular sleep-wake schedule of 8 hours of sleep and 16 hours of wake for at least two weeks, followed by a week of 6 hours sleep per night, timed to end at their habitual wake time, prior to laboratory admission. The sleep times were verified by calls to a time- and date-stamped voicemail and continuous actigraphy monitoring (Actiwatch-L; Minimitter, Inc.). Participants were required to abstain from caffeine, nicotine, alcohol, and other foreign substances for the duration of the study from the beginning of screening until completion, and compliance was evaluated by toxicology tests during screening and upon admission. In addition, female participants were confirmed to be non-pregnant during screening and upon admission. Female participants not using oral contraceptives were tested in the luteal phase of their menstrual cycle (based on menstrual history), and female participants taking oral contraceptive were tested during times when they took a contraceptive with a stable hormone concentration.

### Protocol design

During the 13-day protocol, participants remained in an individual suite free of time cues. Participants did not see or interact with any other study volunteers. On the day of admission (day 0; Fig. 1), subjects woke at their habitual wake time after a six-hour sleep opportunity and reported to the laboratory at noon where they remained in ambient light during admission procedures. Ambient light was provided by 4100 K fluorescent lamps (Philips Lighting, The Netherlands) with digital ballasts (Lutron Electronics Co., Inc, PA) transmitted through a UV-stable filter (Lexan 9030 with prismatic lens, GE Plastics, MA). Light levels were approximately 90 lux at the cornea [0.23 W per m^2^ (~89 lux, ~55 melEDI lux) at 137 cm from the floor in the vertical plane and had a maximum of 0.48 W/m^2^ (~150 lux, 93 melEDI lux) when measured in the horizontal plane at a height of 187 cm anywhere in the room]. Ambient light remained at this intensity for the remainder of the study, except during the blood collection for the dim light melatonin onset (DLMO) assessment, sleep episodes and experimental lighting countermeasures.

Twelve hours after habitual wake time during wake period one, the light was dimmed to ~0.5 lux (0.001 W per m^2^, at 137 cm from the floor in the vertical plane with a maximum <3 lux, 0.01 W per m^2^, at 187 cm from the floor in the horizontal plane anywhere in the room) (grey bars, Fig. 1) and an IV was inserted for blood collection. Participants were in darkness (black bars, Fig. 1) for their scheduled six-hour sleep episode. On wake period two (WP2, day 1) subjects woke at their habitual wake time, had their IV removed, and underwent a final robotics training split in two blocks of three hours each with a three-hour break in between starting two hours after wake (dark gray bars, Fig. 1). Thirteen hours into wake period 2, subjects had a scheduled 3-hour sleep episode before starting their first block of slam-shifting (WP3, day 2, Fig. 1), during which their sleep opportunity was shifted by 9 hours to occur during the daytime in order to mimic conditions for astronauts at the ISS. Testing started 10 hours into wake period 4 (day 3) and lasted for six consecutive hours, followed by an additional one and half hours of time before the next 6-hour sleep episode occurred. After two hours in ambient light on wake period 5 (day 3), the light intensity was dimmed to ~0.5 lux again and another IV insert occurred (hatched bars indicating DLMO collection, Fig. 1). After 9 hours of wakefulness, subjects went to bed at their habitual sleep time and were allowed to sleep for 6 hours. The IV was removed upon waking on wake period 6 (day 4) and another cycle of slam-shifting (WP7, WP8) and robotics testing (WP8) was initiated, identical to the first one described above, which was repeated two more times throughout the protocol (WP12, WP 16) before the subjects were discharged on the evening of day 12 after a final 8-hour recovery sleep.

### Countermeasures

Three countermeasures following one baseline condition were tested consisting of 4100 K white light plus placebo (baseline) and A) 4100 K white light plus caffeine, B) 6500 K blue-enriched white light plus placebo, and C) 6500 K blue-enriched white light plus caffeine. Light Correlated Color Temperatures (CCT) in Kelvins (K) were nominally 4100 K or 6500 K but measured values are shown in Supplemental Fig. 1B. All participants completed the baseline condition first and were then randomly assigned to one of three countermeasure orders; BCA (*n* = 6), ACB (*n* = 6), CBA (*n* = 4). We chose to use three, rather than all six, possible countermeasure orders in an attempt to reduce between-subject sleep-debt effects that we expected to be large, and given the relatively small number of participants available. The original design included a single fixed order for all participants but upon reflection, this was modified before study start to address the limitations of that approach.

Low-dose caffeine (0.3 mg per kg body weight) or placebo was administered orally in pill form by a nurse every hour starting at wake time until 2 hours prior to bedtime during countermeasure testing (WP4, WP8, WP12, WP16).

Experimental light exposures were provided by the Solid-State Lighting Module –Research (SSLM-R). It is a prototype LED lighting system for informing the development of the Solid-State Lighting Assemblies (SSLAs) that were installed on the International Space Station beginning in 2016 to replace the current fluorescent light General Illuminaire Assemblies (GLAs). The SSLM-R was developed at Kennedy Space Center (Bionetics Corporation, Cape Canaveral, FL) and contained LED arrays of 294 white LEDs and 254 RGB LEDs behind a lens diffuser. The SSLM-R was programmed to produce two polychromatic white light spectra with relatively lower (4000 K CCT) or higher (6200 K CCT) short-wavelength light content, and therefore lower or higher melanopic lux, respectively^34,66^. Two sets of SSLM-R panels were mounted on the cart that held a robotic workstation and another set was mounted on a stand, placed behind the seated participants, serving as the outer border of the 2 × 2 m space (see Liu et al.^53^ accompanying manuscript, Fig. 5, panel A). The experimental light exposures were a combination of the LEDs and light from the robotics workstation screens which increased blue light exposure slightly in both conditions. Light intensities with robotics screens on were set to have equal illuminance levels at the cornea at ~90 lux at 137 cm from the floor in the vertical plane for both conditions. The resultant lights were measured at 94 lux, with melanopic EDI lux levels of 88 lux and 71 lux for 6300 K and 4700 K, respectively (Supplemental Fig. 1).

Routine irradiance and illuminance readings were taken using an IL-1400 radiometer with an SEL-033/Y/W detector and SEL-033/F/W detector, respectively (International Light, Inc., Newburyport, MA). Spectrophotometer readings were performed using a PR-650 SpectraColorimeter (PhotoResearch Inc., Chatsworth, CA).

### Outcomes

Each 6-hour test session (dark gray bars, Fig. 1) consisted of four repetitions of a one-hour robotics test block followed by a 30-minute cognitive test block. The 1-hour robotics test block consisted of 10 Track & Capture tasks, 2 “Fly-to and Grapple” tasks, and 2 Autosequence tasks and were presented in the same order in each block (for details please see Liu et al.,^53^ accompanying manuscript). The 30-minute cognitive test block included the visual analogue scale (VAS), the Karolinka Sleepiness Scale (KSS), the Digit Symbol Substitution Task (DSST), the 10-minute visual version of the Psychomotor Vigilance Test (PVT), and the Karolinska Drowsiness Test (KDT). Each of these tests was administered on a standardized testing computer that was used solely for the purpose of administering these tests. The VAS was displayed as a 100 mm line with the anchors “sleepy” and “alert.” Participants could select any point on the line in 1 mm increments. The DSST lasted two minutes and included symbols paired with numbers in a row, with a target symbol displayed in the center of the screen. The participant was instructed to select the number corresponding to the symbol that matched the target as quickly as possible. The PVT was completed using a response box with two buttons. Participants were instructed to watch a box on the computer screen and rest their thumbs on the response box buttons. They were instructed to respond as quickly as possible when numbers began to scroll up by pressing the response box button corresponding to their dominant hand. The inter-stimulus interval for the PVT was set between 2 and 10 seconds, using a rectangular distribution. The KDT is a standardized test whereby the participant was instructed to fixate on a five cm diameter black circle at a distance of one meter.

Melatonin was measured on four occasions during the study (WP1, WP5, WP9, WP13) in order to measure the timing of the circadian system. An indwelling, intravenous catheter was inserted in each participant’s forearm vein and plasma samples were collected every 60 minutes for 12 hours. Samples were analyzed by radioimmunoassay (Buehlmann Laboratories AG, Schoenebuch, Switzerland). DLMO was determined for WP1, WP5, WP9, and WP13 employing a 5 pg per ml threshold criterion. Three participants had melatonin values above the 5 pg per mL threshold. For these individuals a 15 pg/mL threshold was used for DLMO.

Waking EEG data were recorded continuously during the four countermeasure sessions at WP4, WP8, WP12, and WP16 using a portable, modular, battery-operated, ambulatory, digital polysomnographic recorder (Vitaport-3 digital recorder, TEMEC Instruments B.V., Kerkrade, The Netherlands). To minimize the number of electrode applications and removals that participants would have to endure, we used the same montage for both wake and sleep recordings. Recordings consisted of electroencephalogram (EEG), electrooculogram (EOG), chin electromyogram (EMG), and a two-lead electrocardiogram (EKG). Electrodes were positioned according to the International 10-20 system placed according to the American Academy of Sleep Medicine (AASM) recommended EEG montage (F4-M1, C4-M1, although not required by AASM, P4-M1 was also recorded and computed for KDT spectral analysis, and O2-M1^67^). All EEG signals were high-pass filtered (time constant: 1.0 seconds), low-pass filtered (-6 dB at 30 Hz, 12 dB/octave), and digitized (resolution: 12-bit, sampling rate: 256 Hz, storage rate: 256 Hz). The raw signals were stored on a Flash RAM card and downloaded offline. KDT data were analyzed using C4_M1-P4_M1. Impedances were routinely checked and documented prior to and after all recordings to ensure that they were < 10 KOhms.

Waking EEG signals obtained during the KDT were visually inspected in two-second increments to remove artifacts (e.g., sweat, blinks, eye movements, and muscle artifact). All artifact-free epochs were processed using a fast-Fourier Transform (FFT) routine (TEMEC technologies B.V., Heerlen, The Netherlands) with a 10% cosine-tapered window.

Single frequencies were selected a priori for delta (2.5 Hz), theta (5.0 Hz), alpha (9.0 Hz), and high alpha (12 Hz). For completeness, the entire range of power density for 1–20 Hz by condition are provided in Supplemental Fig. 2.

Sleep episodes were scored according to the AASM criteria. The sleep periods following the first three countermeasure administrations lasted six hours. However, the sleep period following the final countermeasure administration (sleep period 16) lasted two hours longer than the other sleep episodes, to provide participants with additional sleep before leaving the laboratory. As a result, analysis of sleep period 16 was limited to the first six hours. The percentage of each stage (wake, stage 1, stage 2, SWS, REM) per sleep episode was assessed to identify differences between sleep following the baseline condition and sleep following the countermeasures.

### Data analysis and statistics

Participants and the investigators were blinded to the randomization of treatments (except for E.E.F. and M.R. who supervised the lighting interventions), and all data analyses were conducted (by A.N.) before the unblinding of experimental conditions. We limited our evaluation to the cognitive test batteries that occurred in conjunction with the timing of the robotics simulation, beginning approximately 10 hours after waking, until approximately 16 hours after waking (Fig. 1). We also restricted our analysis to tests that occurred after an individual’s timing of DLMO in order remove the influence of the circadian wake-maintenance zone in our analyses. Less than 4% of the data were excluded. Given the number of participants that we studied, participant data are not equally represented in each countermeasure condition and test block. To meet Fisher’s criteria, the KDT data were log-transformed, PVT reaction time was transformed to its inverse, the DSST number correct was transformed to its square root and VAS was arcsine transformed. We evaluated the impact of treatment (4700 K light + placebo [baseline], 4700 K light + caffeine, 6300 K light + placebo, 6300 K light + caffeine) on all outcome variables using a mixed-effects regression model adjusted for group order that included subject intercept as a random effect. Mixed-effects models control for the influence of repeated measures among individuals when the subject is included as a random effect. Each of the outcome variables, PVT, DSST, VAS, KSS and the EEG spectra in the delta, theta and alpha regions during the KDT, were evaluated in separate models. The final model for each outcome yielded normally distributed residuals, confirmed by Levene’s test that were homoscedastic across equal, consecutive slices of the range predictions. Effect sizes for all comparisons were calculated using Hedges’ *g* and were adjusted to reduce sample size bias as follows:

1 $$g=\frac{{M}_{1}-{M}_{2}}{{{SD}}_{{pooled}}^{* }}\times \left(\frac{N-3}{N-2.25}\right)\times \sqrt{\frac{N-2}{N}}$$

All analyses were completed using SAS (version 9.4, Cary, NC) and SYSTAT software (SYSTAT Corp oration, Chicago, Ill).

### Supplementary information

Supplemental Material

## Data Availability

De-identified individual data for all outcomes described are available upon request from the corresponding author, subject to appropriate ethical approval and completion of a Data Sharing Agreement. Data will be provided immediately upon completion of these conditions.

## Code availability

Code for all outcomes described are available upon request from the corresponding author, subject to appropriate ethical approval and completion of a Data Sharing Agreement. Code will be provided immediately upon completion of these conditions.
