# Supplementary material for: Effectiveness of caffeine and blue-enriched light on cognitive performance and electroencephalography correlates of alertness in a spaceflight robotics simulation
Source: NPJ Microgravity. 2023 Dec 19;9:93. doi: 10.1038/s41526-023-00332-w (PMC10730879; doi:10.1038/s41526-023-00332-w)
Supplement: Supplementary file 1 — Supplemental Material [file 41526_2023_332_MOESM1_ESM.pdf]

a

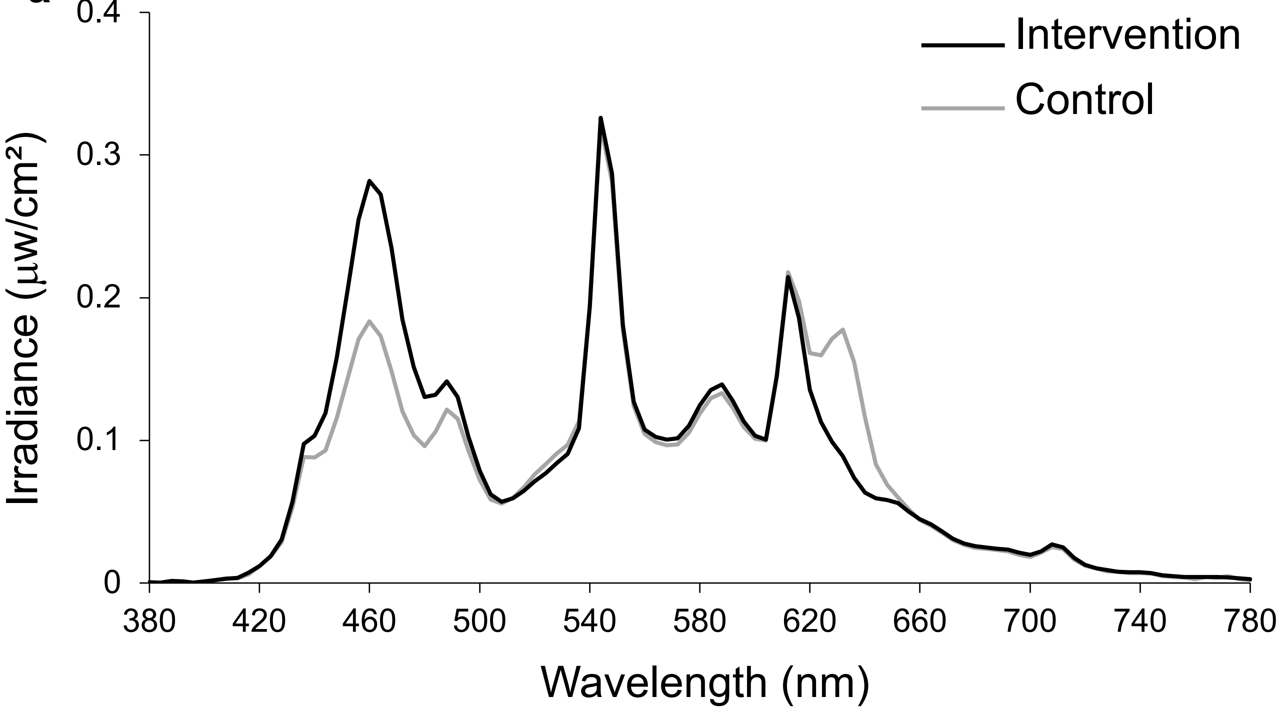

b

	CCT K	Photopic lux	S-cone-opic α-opic EDI (lux)	M-cone-opic α-opic EDI (lux)	L-cone-opic α-opic EDI (lux)	Rhodopic α-opic EDI (lux)	Melanopic α-opic EDI (lux)	Melanopic DER
**Intervention**	6289	93.9	93.5	91.4	93.7	87.2	88.0	0.94
**Control**	4694	94.2	67.6	86.6	93.8	74.8	71.2	0.76

Supplemental Figure 1. a) Spectral power distribution of the Intervention and Control lighting with robotics simulation screen on. b) Experimental light exposure measured at a height of 54" in the vertical plane during simulation including Correlated Color Temperature (CCT, Kelvins), irradiance, photon flux, photopic illuminance (lux) and the corresponding human retinal photoreceptor weighted α-opic equivalent daylight (D65) illuminances (α-opic EDI) and the melanopic Daylight Equivalent Ratio (DER) calculated according to CIE S 026/E:2018.

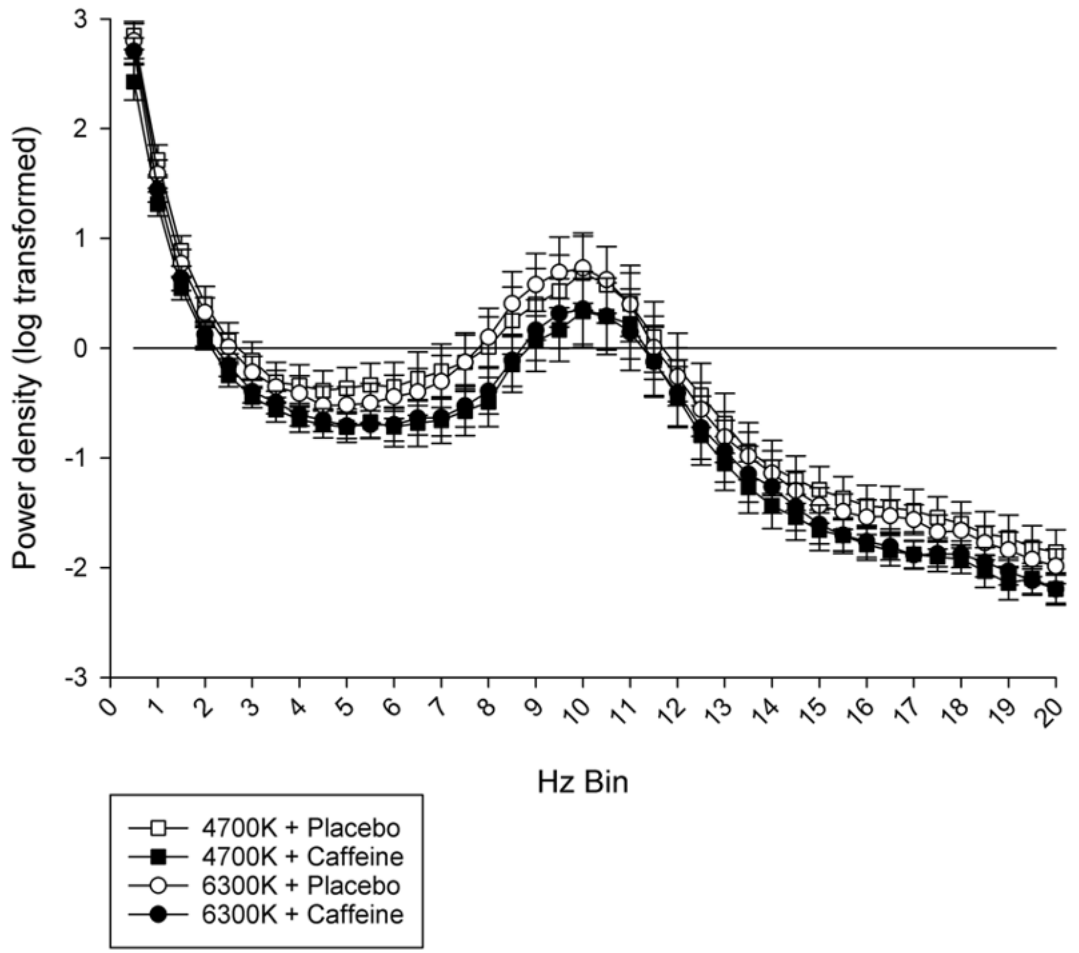

Supplemental Figure 2: Power densities for EEG frequency bins between 1-20 Hz by condition.
